# Antibacterial Activity of Fructose-Stabilized Silver Nanoparticles Produced by Direct Current Atmospheric Pressure Glow Discharge towards Quarantine Pests

**DOI:** 10.3390/nano8100751

**Published:** 2018-09-21

**Authors:** Anna Dzimitrowicz, Agata Motyka-Pomagruk, Piotr Cyganowski, Weronika Babinska, Dominik Terefinko, Piotr Jamroz, Ewa Lojkowska, Pawel Pohl, Wojciech Sledz

**Affiliations:** 1 Department of Analytical Chemistry and Chemical Metallurgy, Faculty of Chemistry, Wroclaw University of Science and Technology, Wybrzeze St. Wyspianskiego 27, 50-370 Wroclaw, Poland; anna.dzimitrowicz@pwr.edu.pl (A.D.); dominik.terefinko@pwr.edu.pl (D.T.); piotr.jamroz@pwr.edu.pl (P.J.); pawel.pohl@pwr.edu.pl (P.P.); 2 Department of Biotechnology, Intercollegiate Faculty of Biotechnology University of Gdansk and Medical University of Gdansk, Abrahama 58, 80-307 Gdansk, Poland; agata.motyka@biotech.ug.edu.pl (A.M.-P.); weronikababinska29@gmail.com (W.B.); ewa.lojkowska@biotech.ug.edu.pl (E.L.); 3 Department of Polymer and Carbonaceous Materials, Faculty of Chemistry, Wroclaw University of Science and Technology, Wybrzeze St. Wyspianskiego 27, 50-370 Wroclaw, Poland; piotr.cyganowski@pwr.edu.pl

**Keywords:** atmospheric pressure plasma, nanostructures, phytopathogens, plant protection, quarantine, *Erwinia amylovora*, *Clavibacter michiganensis*, *Ralstonia solanacearum*, *Xanthomonas campestris* pv. *campestris*, *Dickeya solani*

## Abstract

Development of efficient plant protection methods against bacterial phytopathogens subjected to compulsory control procedures under international legislation is of the highest concern having in mind expensiveness of enforced quarantine measures and threat of the infection spread in disease-free regions. In this study, fructose-stabilized silver nanoparticles (FRU-AgNPs) were produced using direct current atmospheric pressure glow discharge (dc-APGD) generated between the surface of a flowing liquid anode (FLA) solution and a pin-type tungsten cathode in a continuous flow reaction-discharge system. Resultant spherical and stable in time FRU-AgNPs exhibited average sizes of 14.9 ± 7.9 nm and 15.7 ± 2.0 nm, as assessed by transmission electron microscopy (TEM) and dynamic light scattering (DLS), respectively. Energy dispersive X-ray spectroscopy (EDX) analysis revealed that the obtained nanomaterial was composed of Ag while selected area electron diffraction (SAED) indicated that FRU-AgNPs had the face-centered cubic crystalline structure. The fabricated FRU-AgNPs show antibacterial properties against *Erwinia amylovora*, *Clavibacter michiganensis*, *Ralstonia solanacearum*, *Xanthomonas campestris* pv. *campestris* and *Dickeya solani* strains with minimal inhibitory concentrations (MICs) of 1.64 to 13.1 mg L^−1^ and minimal bactericidal concentrations (MBCs) from 3.29 to 26.3 mg L^−1^. Application of FRU-AgNPs might increase the repertoire of available control procedures against most devastating phytopathogens and as a result successfully limit their agricultural impact.

## 1. Introduction

In recent decades, a rapid increase in the fabrication of noble metal nanoparticles (NPs) has been observed. Dynamic development in production of these inorganic nanostructures has been associated with their unique optical [1], chemical [2], and photothermal [3] properties, which are different from those of bulky samples made of the same material. Among noble metal nanostructures, the most studied and utilized are silver nanoparticles (AgNPs). Various methods have been developed for synthesis of AgNPs so far [4,5]. The most common are based on chemical [6], physicochemical [7], and biological [8] reduction of Ag(I) ions. Importantly, these methods are usually multi-step and, hence, time-consuming. Additionally, the chemical reduction approach often requires toxic reagents such as hydrazine [9]. For these reasons, several research groups applied atmospheric pressure plasmas (APPs) generated in contact with liquids and tried to use plasma-liquid interactions (PLIs) for the synthesis of stable in time AgNPs in a much faster and less complicated way [10,11,12,13,14,15,16,17]. In our group, several continuous-flow reaction-discharge systems based either on the operation of dc-APGD [18,19] or a pulse modified radio frequency version of this discharge (pm-rf-APGD) [20] were developed and used for the fabrication of AgNPs. In these systems, APP was operated between a flowing liquid electrode, which were the solutions of the AgNPs precursor and certain stabilizers and a pin-type solid tungsten electrode. The operation of APP resulted in the production of various reactive oxygen and nitrogen species (RONS) in addition to solvated electrons and hydrogen radicals (H^∙^). Subsequently, all these species mediated reduction of Ag(I) ions and formation of AgNPs [18,19,20].

AgNPs have found many interesting applications for instance in degradation of organic dyes in catalysis [21], ultrasensitive DNA detection in biosensors [22], inactivation of bacteria in textile production [23], and treatment of human melanoma cancer in medicine [20]. Additionally, their possible implementation into agriculture has been suggested previously [18,24,25]. Having in mind synthesis of biocompatible, natural, and low-cost nanomaterials [26,27] for such a practice, several AgNPs synthesis methods based on reductive properties of plant extracts [28], natural biopolymers [29], agricultural wastes [30], or commercially available biocontrol agents [31] have been developed up to the present day. Importantly, AgNPs might be efficiently applied for direct eradication of fungal [32,33,34,35] and bacterial [18,24,25] phytopathogens. Considering that various formulations of fungicides are still successful in plant disease control [36] while costs of quarantine enforcement are quite high, the objective of the present study was to investigate the activity of plasma synthesized AgNPs against bacterial phytopathogens subjected to compulsory control measures, according to European Union legislation by a Council Directive 2000/29/EC and posterior directives (most recently Commission Implementing Directive 2017/1279) amending its annexes. For this research, we focused on *Erwinia amylovora* (Eam), *Clavibacter michiganensis* (Cm), *Ralstonia solanacearum* (Rsol), *Xanthomonas campestris* pv. *campestris* (Xcc), and *Dickeya solani* (Dsol) whose economic significance was further emphasized by either a place or honorable mentions on the top 10 list of plant pathogenic bacteria by Mansfield et al. [37].

Eam is a Gram(−) bacterial phytopathogen that causes fire blight on the *Rosaceae* family including mostly subfamilies *Maloideae* or *Pomoideae* [37]. Typical symptoms of the above-mentioned disease include flower necrosis, fruit rot, shepherd’s crook in shoots, bacterial ooze, and cankers in woody tissue [38]. The highest economic losses are recorded on apples and pears and might result in disrupting orchard production for several years. To exemplify, the financial impact of Eam recorded in the north-west part of USA exceeded 68 million dollars in 1998 alone [39]. On the other hand, Cm of Gram(+) coryneform morphology is responsible for bacterial canker on tomato. Most recognizable diagnostic symptoms of this disorder involve wilting and bird’s eye-spot lesions among less species-specific, but highly devastating being vascular discolorations, brown streaks opening as cankers, leaf necrosis, plant stunting and desiccation in addition to premature fruits fall. Substantial financial damage in tomato production might even reach $300,000 per grower in a single year [40]. On the contrary, Rsol is a Gram(−) soil-borne causative agent of bacterial wilt on several hundred plant species belonging to more than 44 families [41]. Notably, plant-specific names of this disease are frequently used, i.e., brown rot on potato and Moko disease on banana. The pathogen penetrating from soil into plant roots reaches the xylem where it multiplies and subsequently triggers systemic infection. Plants wilt, their stems soften and split while releasing bacterial exudates. Focusing on potatoes, browning of the tuber vascular ring characterizes an advanced stage of Rsol infection. High economic impact of this disease mainly results from wide geographic distribution and costly quarantine procedures. For instance, about 50% potato tuber losses caused by these bacteria are noted in India [42]. Concerning an etiological agent of black rot being a Gram(−) rod Xcc, its most important hosts include the members of the crucifer family *Brassicaceae* with cabbage, cauliflower, broccoli, radish, Brussels sprouts, and kale [43]. In the case of this disorder, contaminated plant tissues become necrotic and leaves fall prematurely while severe rotting leading to plant death follows systemic infection. V-shaped, chlorotic yellow lesions are typical symptoms. Black rot was detected on all continents and it is regarded as the most important disease of brassica worldwide [44]. Dsol, which is a Gram(−) rod-shaped bacterium not yet regarded as a quarantine microorganism outside Israel and North Africa countries, was subjected to a zero tolerance policy in Scotland [45]. This relatively new threat to European potato production has been efficiently spreading across the continent since 2005 [46]. Dsol causes blackening and softening of the stem base referred to as blackleg in addition to soft rot meaning maceration and collapse of the inner tuber tissue. Interestingly, the resultant disease symptoms besides their severity are indistinguishable from these caused by other species from the genera *Dickeya* or *Pectobacterium*. To illustrate, Tsror et al. [47] reported potato yield reduction of 20% to 25% when the disease incidence exceeded 15%.

Here, the dc-APGD-based continuous-flow reaction-discharge system was applied to produce uniform and monodisperse spherical AgNPs stabilized by fructose (FRU). Optical properties of the synthesized FRU-AgNPs were analyzed by using UV/Vis absorption spectrophotometry. Their granulometric properties were examined with the aid of transmission electron microscopy (TEM) supported by energy dispersive X-ray spectroscopy (EDX) and selected area electron diffraction (SAED). Dynamic light scattering (DLS) was further used to evaluate the size of the produced Ag nanostructures. To confirm surface functionalization of AgNPs by FRU, attenuated total Reflection-Fourier transformation infrared spectroscopy (ATR FT-IR) was applied. Lastly, antibacterial properties of the resultant FRU-AgNPs were studied against phytopathogenic microorganisms classified to Eam, Cm, Rsol, Xcc, and Dsol species.

## 2. Materials and Methods

### 2.1. Reagents and Solutions

A working solution of the AgNPs precursor (100 mg L^−1^ of Ag(I) ions with 0.25% (*m*/*v*) d-fructose) was prepared as follows: 0.0157 g of solid silver nitrate (AgNO_3_, Avantor Performance Materials, Gliwice, Poland) and 0.25 g of d-fructose (Avantor Performance Materials, Gliwice, Poland) were dissolved in water. The concentration of the capping agent (0.25% (*m*/*v*) of FRU) in this solution was chosen in order to allow for stable operation of dc-APGD in the continuous-flow reaction-discharge system. All reagents were of analytical grade or higher purity. Re-distilled water was used throughout.

### 2.2. Production of FRU-AgNPs in the dc-APGD-based Reaction-Discharge System

**Figure 1:**
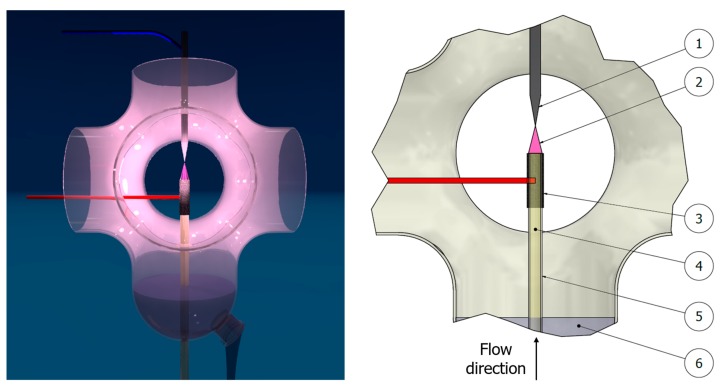
A schematic representation of the dc-APGD-based reaction-discharge system working in a continuous-flow mode. (1) A pin-type W cathode, (2) dc-APGD, (3) a graphite tube, (4) a working solution (with the AgNPs precursor and d-fructose) acting as a flowing liquid anode (FLA), (5) a quartz capillary, and (6) a compartment for the collection of the dc-APGD treated working solution containing the synthesized FRU-AgNPs.

FRU-AgNPs were synthesized in the dc-APGD-based continuous-flow reaction-discharge system previously described by Dzimitrowicz et al. [19]. The working solution of the AgNPs precursor was introduced to the system through a quartz capillary (OD = 4.0 mm, ID = 2.0 mm) onto which a graphite tube (OD = 6.0 mm, ID = 4.0 mm) was mounted (Figure 1). The flow rate of this solution was 2.0 mL min^−1^ and was maintained by applying a four-channel peristaltic pump (Masterflex L/S, Cole-Parmer, Vernon Hills, IL, USA). In these conditions, the working solution acted as the flowing liquid anode (FLA) while a solid tungsten (W) electrode was the cathode of this discharge system. dc-APGD was sustained and stably operated between the surface of this FLA solution and the sharpened tip of the W cathode (ID = 4.0 mm). Both electrodes were placed inside a 90 mm (height) by 40 mm (radial) quartz chamber. The distance between them was 5.0 mm to allow for stable operation of dc-APGD. To ignite dc-APGD, a high voltage (HV) of 1100–1300 V provided by a dc-HV supplier (Dora Electronics Equipment, Wroclaw, Poland) was supplied to both electrodes. Stabilization of the discharge current (30 mA) was maintained by applying a ballast resistor of 10 kΩ (Tyco Electronics, Berwyn, IL, USA) situated in the anode circuit. The dc-APGD-treated working solution, which contained the synthesized FRU-AgNPs, was collected into 10 mL glass vials and kept for further analyses including determination of their optical, granulometric, and antibacterial properties.

### 2.3. Characterization of Optical and Granulometric Properties of FRU-AgNPs

To examine suitability of dc-APGD for the production of stable in time, spherical, monodisperse, and uniform in size FRU-AgNPs, their optical and granulometric properties were evaluated.

Optical properties of the produced Ag nanostructures were assessed by UV/Vis absorption spectrophotometry. UV/Vis absorption spectra were acquired by using a Specord 210 spectrophotometer (Analytik Jena AG, Jena, Germany) in the spectral range from 300 nm to 900 nm. The scanning speed was 20 nm s^−1^ and the step was 1 nm.

Granulometric properties (size, shape, elemental composition, and crystalline structure) of the fabricated FRU-AgNPs were determined by TEM (Tecnai G^2^20 X-TWIN, FEI, Hillsboro, OR, USA). The measurements were performed in a bright field and in electron diffraction modes for direct imagining and SAED, respectively. For EDX analyses, the mode of energy dispersion of X-rays supported by an EDX detector was applied. To carry out all analyses according to the granulometric properties of the resultant FRU-AgNPs, one-drop of the dc-APGD-treated working solution was placed onto an ultra-thin carbon-copper grid (CF400-Cu-UL, Electron Microscopy Sciences, Hatfield, PA, USA). Then, the so-prepared sample was sequentially rinsed with re-distilled water and dried to remove sugar from it before the analysis. To analyze the collected data, the FEI software (version 3.2 SP6 build 421, FEI, Hillsboro, OR, USA) was applied. The size distribution of the FRU-AgNPs was determined from high-resolution TEM photomicrographs. Since removal of FRU resulted in the creation of a considerable number of agglomerates, the graphics were segmented by applying image thresholds that allow the detection of a single nanoparticle within an agglomerate. Then, the counted particles were analyzed by using Microsoft Excel (Richmond, VA, USA) Analysis Tool Pack add-in, which created an appropriate histogram. Size distribution by the number of FRU-AgNPs was also estimated by DLS and by applying a Photocor Complex instrument (Photocor Instruments, Tallin, Estonia) equipped with a 638 nm/25 mW^3^ laser. Measurements were performed in round glass vials (ID = 14.8 mm) submerged in decalin at the scattering angle of 90°. Temperature during all tests was 21.96 °C and water viscosity was 0.9864 mPa·s^−1^. DynaLS software (Alango Ltd., Tirat Carmel, Israel) was utilized for data evaluation.

### 2.4. Surface Functionalization of AgNPs by FRU

To confirm the stabilizing role of FRU during AgNPs production, ATR FT-IR spectroscopy was applied. Respective ATR FT-IR spectra were acquired for two samples: (i) the raw working solution prior to treatment with dc-APGD and (ii) the dc-APGD-treated working solution containing the produced FRU-AgNPs. All ATR FT-IR spectra were collected in the range from 4000 to 400 cm^−1^ using a Nicolet 6700 instrument (Thermo Fisher Scientific, Waltham, MA, USA) that was equipped with a Smart Orbit ATR accessory. Measurements were taken at a resolution of 4 cm^−1^ and the scans number was 64. All analyses were carried out under vacuum conditions.

### 2.5. Purification of FRU-AgNPs

In order to purify the fabricated FRU-AgNPs from unreacted Ag(I) ions, dialysis, as previously reported by Dzimitrowicz et al. [18], was used. A portion of the dc-APGD-treated working solution with the synthesized FRU-AgNPs was poured into a dialysis tube with a molecular weight cut-off of 14,000 Da (Sigma-Aldrich, Poznan, Poland) and immersed in 500 mL of re-distilled water in a glass beaker. Then, the glass beaker was placed onto a magnetic laboratory stirring plate (WIGO, Pruszkow, Poland) and its contents was subjected to stirring at 1000 rpm for 24 h.

### 2.6. Determination of the Concentration of the Purified FRU-AgNPs

To determine the concentration of the purified FRU-AgNPs (as Ag) after dialysis, flame atomic absorption spectrometry (FAAS) was used. The appropriate volume of the solution obtained after dialysis from the dialysis tube was poured into a 200-mL beaker and treated with a 65% (m/m) HNO_3_ solution (Avantor Performance Materials, Gliwice, Poland). Afterwards, the resulting mixture was heated to boil for 30 min on a hot plate for digestion of FRU-AgNPs. Next, a PerkinElmer (Bodenseewerk Perkin-Elmer GmbH, Uberlingen, Germany) single-beam FAAS instrument, model 1100B with a deuterium lamp, was applied for quantification of the Ag concentration in the final sample solution. 

### 2.7. Bacterial Strains and Their Culture Methods

Plant pathogenic bacteria investigated in this study are listed in Table 1. All microorganisms originated from the collection of bacterial phytopathogens of the Intercollegiate Faculty of Biotechnology University of Gdansk and Medical University of Gdansk (IFB UG & MUG) (Gdansk, Poland) and had been previously stored at −80 °C in 40% (*v*/*v*) glycerol. The tested microorganisms were recovered from frozen stocks by plating on optimal solid media (Table 1). 24 h of incubation at 28 °C followed. To obtain the overnight liquid cultures, a single bacterial colony per species was utilized for the inoculation of the proper liquid medium (Table 1) prior to 24 h of incubation at 28 °C.

### 2.8. Antibacterial Properties of FRU-AgNPs Against Bacterial Phytopathogens

Overnight liquid bacterial cultures were centrifuged for 10 min at 6000 rpm. The harvested cells were washed twice and subsequently suspended in a sterile 0.85% NaCl solution to reach the turbidity of 0.5 in the McFarland scale (McF) as measured by a DEN-1B densitometer (BioSan, Riga, Latvia). The purified FRU-AgNPs were dissolved in sterile re-distilled water to obtain 2×, 3×, 4×, 6×, 8×, 16×, and 32× dilutions. 10 µL of the 0.5 McF bacterial suspensions, 90 µL of the corresponding growth media (Table 1), and 100 µL of FRU-AgNPs dilutions or the concentrated stock solution were added to each well in sterile 96-well microplates. Appropriate negative controls (containing solely the respective growth media or these media supplemented with 0.85% NaCl or re-distilled water) and positive controls (the respective growth media inoculated with a given phytopathogen) were included. Optical densities at 600 nm (OD_600_) of bacterial cultures within the microplates were measured by using an EnVision Multilabel Plate Reader (PerkinElmer, Waltham, MA, USA). Incubation at 28 °C for 24 h followed. Then, OD_600_ of the bacterial cultures were investigated again to state minimal inhibitory concentrations (MICs) of FRU-AgNPs to define the concentration of AgNPs potent enough to inhibit the growth of bacterial phytopathogens in liquid media [18,55]. The contents of 96-well microplates showing no visible bacterial growth were also plated on the appropriate solid growth media (Table 1) to establish minimal bactericidal concentrations (MBCs) as described previously [18,55]. The plates were incubated at 28 °C for 24 h. The resultant bacterial colonies were counted. The whole experiment was repeated in triplicate for each bacterial strain.

## 3. Results and Discussion

### 3.1. Optical Properties of FRU-AgNPs

**Figure 2:**
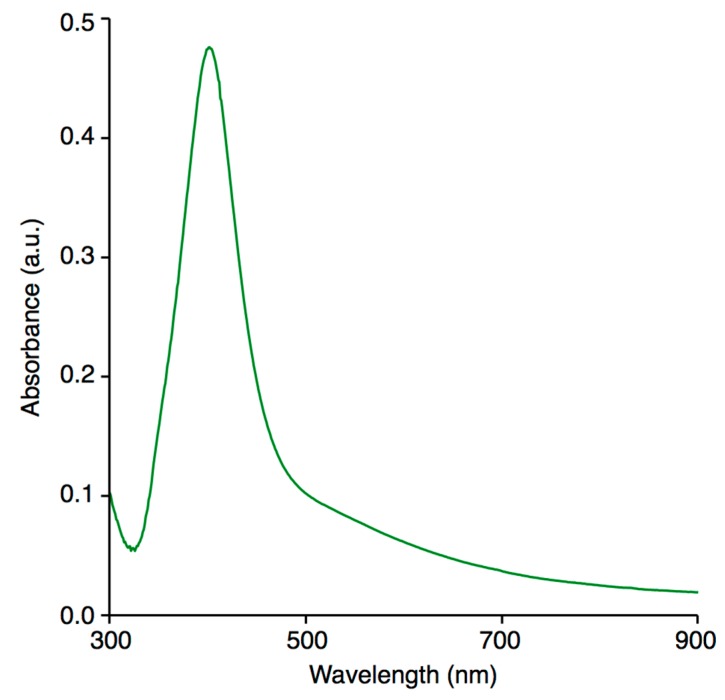
The UV/Vis absorption spectrum of five times diluted colloidal suspension of FRU-AgNPs.

It was possible to confirm the formation of Ag nanostructures as well as to estimate their optical properties based on UV/Vis absorption spectra of the dc-APGD-treated working solution (Figure 2). Spherical metallic nanostructures of different sizes are able to absorb and reflect light of unique wavelengths, which results in localized surface plasmon resonance (LSPR) absorption bands of different widths and centered around different wavelengths due to mutual vibration of their free electrons in resonance with given light waves [56]. The LSPR absorption band for small spherical AgNPs is typically situated between 380 to 450 nm [57]. In the present contribution, the UV/Vis absorption spectrum of the dc-APGD treated working solution, which was dominated by the LSPR absorption band with wavelength at its maximum at 404 nm (Figure 2). This confirms the formation of spherical FRU-AgNPs. Additionally, the symmetrical shape of this LSPR absorption band and a low value of its full width at half maximum (FWHM), i.e., 79 nm, suggested that the resultant spherical FRU-AgNPs were monodisperse and non-aggregated (Figure 2) [58].

### 3.2. Granulometric Properties of FRU-AgNPs

**Figure 3:**
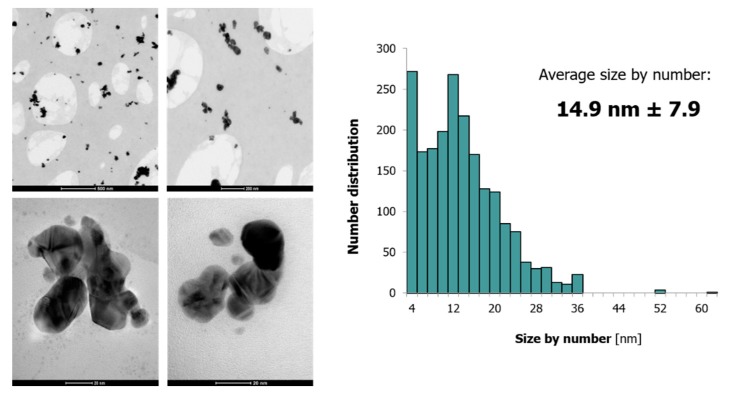
TEM micrographs illustrating shapes and size distribution of FRU-AgNPs.

Using TEM supported by SAED and EDX as well as DLS, granulometric properties of the fabricated FRU-AgNPs according to their size, shape, elemental composition, and crystalline structure were assessed. Based on TEM measurements, it was established that FRU-AgNPs were approximately spherical (95%) even though the structures of other shapes, i.e., triangular and hexagonal, were also detected (Figure 3). The average size of the resultant FRU-AgNPs along with its size distribution was 14.9 ± 7.9 nm, which means that they were quite uniform with a narrow size distribution (Figure 3). FRU-AgNPs were well dispersed in the aqueous medium, but several aggregates were also observed (Figure 3). The occurrence of aggregates might be related to the sample preparation for TEM measurements (see Section 2.3 for more details).

**Figure 4:**
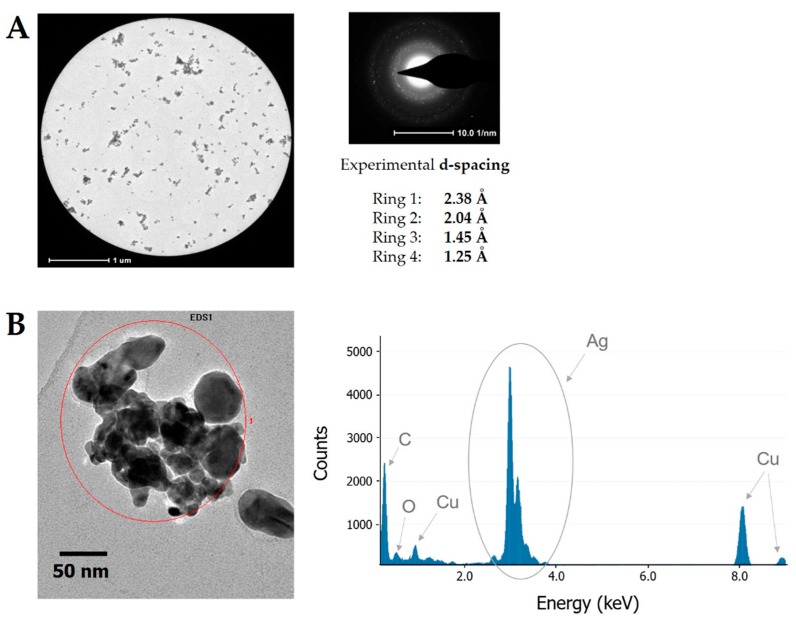
Granulometric properties of Ag nanostructures (**A**) The SAED pattern for the micrograph of FRU-AgNPs and (**B**) the EDX spectrum for the presented FRU-AgNPs.

Figure 4A presents the SAED pattern of the synthesized Ag nanostructures. Based on this, corresponding rings associated with the face-centered cubic (fcc) highly crystalline structure of the produced FRU-AgNPs were determined. Observed d-spacings were 2.38, 2.04, 1.45, and 1.25 Å, which indicates Miller indices of (111), (200), (220), and (311), respectively [18]. To reveal the elemental composition of the synthesized nanomaterial, EDX was applied. Figure 4B shows the respective EDX spectrum. The following elements were identified: Ag (from the synthesized AgNPs), O and C (both from the chemical structure of FRU), and Cu (from the grid onto which the APP-treated working solution was deposited).

**Figure 5:**
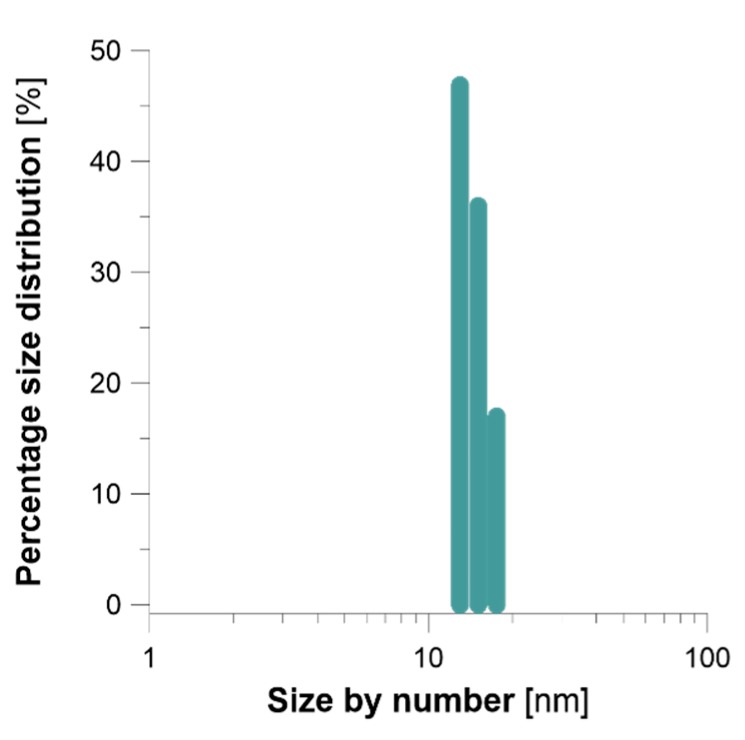
Percentage size distribution by the number of FRU-AgNPs estimated by dynamic light scattering (DLS).

DLS analyses were performed to corroborate size distribution of FRU-AgNPs calculated on the basis of TEM imaging. Figure 5 presents percentage size distribution by the number of resultant FRU-AgNPs obtained after treatment of the working solution in the applied, dc-APGD-based, continuous-flow reaction-discharge system. Average size of FRU-AgNPs and its distribution was 15.7 ± 2.0 nm and was slightly larger than was determined by using TEM. This discrepancy in the average size, as determined by TEM and DLS, is commonly reported in literature [59]. By applying TEM, it was possible to accurately evaluate the size of the metal core of NPs when compared to DLS in which light is scattered on the analyzed nanomaterial.

The above-listed measurements proved that, by applying dc-APGD operated between the surface of the FLA solution and the pin-type W cathode in the utilized continuous-flow reaction-discharge system, it was possible to obtain uniform and approximately spherical FRU-AgNPs with a narrow size distribution. The proposed system produced 120 mL of colloidal suspensions of FRU-AgNPs per hour. This certainly overcame limitations related to insufficient yield of NPs reported in the case of stationary reaction-discharge systems in which APPs also operated in contact with liquids [10,11,12,13,14,15,16,17]. Subsequently, FAAS was used to determine the concentrations of the purified solutions by dialysis with FRU-AgNPs. The Ag concentration of 52.6 mg∙L^−1^ was assessed in the working solution after its dc-APGD treatment and posterior further purification. Considering that the initial concentration of Ag(I) ions in the working solution was 100 mg∙L^−1^, it gave 52.6% efficiency of FRU-AgNPs production by the proposed APP-based system. 

### 3.3. Stabilization of the Surface of AgNPs by FRU

**Figure 6:**
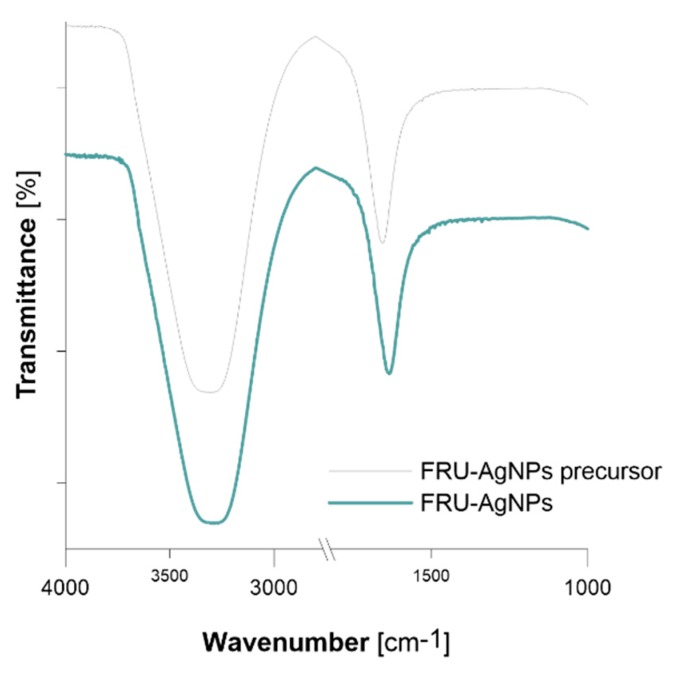
ATR FT-IR spectra of the working solution before (FRU-AgNPs precursor) and after (FRU-AgNPs) dc-APGD treatment.

Efficacy of AgNPs surface stabilization by FRU was examined on the basis of ATR-FTIR spectra of the working solution before and after dc-APGD treatment (Figure 6). For the working solution after dc-APGD treatment, strong absorption bands at 3279 cm^−1^ and 1656 cm^−1^ attributed to ν stretching vibrations of the −O−H group and ν stretching vibrations of the C=O group present in FRU were detected [60]. Slight shifts in the position of strong absorption bands (about 21 cm^−1^) could be seen when comparing ATR FT-IR spectra of the working solution before and after dc-APGD treatment. Such band shifts might point an interaction of the surface of AgNPs with FRU, which confirms its stabilizing role. Furthermore, the chemical structure of FRU contains a cyclic ring with several −OH groups. The possibility of hydrogen bonding formation between FRU and the surface of Ag nanostructures accounts for efficient application of this monosaccharide as a stabilizer for preventing uncontrolled growth, aggregation, and sedimentation of AgNPs.

The further advantage of FRU utilized as a stabilizer is related to its classification as an eco-friendly green capping agent [61]. FRU was previously applied in APP-based synthesis of AgNPs by several research groups [11,16,17]. Richmonds and Sankaran [11] reported a reaction-discharge system with an atmospheric-pressure microplasma (APM) cathode operated in an H-shaped glass cell between the surface of a solution and an Ar nozzle jet [11]. The size of the obtained FRU-AgNPs was 10 nm [11]. Kondeti et al. [16] used a radio-frequency driven APP jet for the production of either raw-AgNPs or FRU-AgNPs. It was found that FRU could be not only the AgNPs-stabilizing surfactant but also the OH scavenger that acted as a reducing agent through the formation of a respective aldehyde and the removal of OH (and H) radicals produced by APP [16]. Chiang et al. [17] also synthesized small FRU-AgNPs (size circa 10 nm) by applying a hybrid microplasma-based electrochemical cell operated at ambient conditions. Importantly for our research, FRU was proven to trigger chemotaxis of plant pathogenic bacteria [62]. Therefore, having in mind future agricultural applications of AgNPs synthesized by the dc-APGD-based reaction-discharge system, this monosugar was selected for the described research and surface stabilization on the fabricated spherical Ag nanostructures.

### 3.4. Antibacterial Properties of FRU-AgNPs against Bacterial Phytopathogens

Up to the present day, Ag nanostructures have been shown to efficiently inhibit the growth of fungal phytopathogens (such as *Bipolaris sorokiniana*, *Magna porthegrisea* [32], *Colletotrichum* spp. [33], *Fusarium oxysporum* [34], *Alternaria alternata*, *Sclerotinia sclerotiorum*, *Macrophomina phaseolina*, *Rhizoctonia solani*, *Botrytis cinerea*, *Curvularia lunata* [35]) and bacterial phytopathogens (for instance, *Pseudomonas syringae* pv. *syringae*, *Xanthomonas campestris* pv. *vesicatoria* [25], *Xanthomonas perforans* [24], *Pectobacterium* and *Dickeya* spp. [18]). Notably, greater interest has been given to the potential application of AgNPs for the management of plant diseases of fungal origin rather than bacterial, which is putatively due to the necessity of higher effective concentrations [25] or more complex nanocomposites [24] to inactivate the latter phytopathogenic microorganisms. The effectiveness of AgNPs application was demonstrated in greenhouses [24], under field conditions [25] in addition to multiple laboratory screenings. These findings encouraged our group to examine antimicrobial potency of the synthesized FRU-AgNPs against plant pathogenic bacteria subjected to compulsory control measures. Quarantine pests spread efficiently and cause considerable financial damage not only to potato breeding companies but also to single growers. Surveillance for the presence of quarantine microorganisms is compulsory and is conducted by the governmental inspectors. Commonly, besides the destruction of the affected plantation (even entire orchards in the case of Eam), specific monitoring procedures with buffer zones around the infested areas are implemented in addition to raising public awareness by running national and international campaigns. In addition, special warning systems based on climatic data are being developed. For instance, according to the European and Mediterranean Plant Protection Organization (EPPO), in the case of the detection of Eam, an integrated program of chemical control combined with sanitation, pruning, eradication, tree nutrition, and planting of resistant or tolerant cultivars is recommended [63]. Regarding direct control, since streptomycin sprays that suppressed Eam in the USA are not allowed for agricultural use in the European Union, several other chemicals such as flumequine, kasugamycin, fosetyl-Al, or oxonilic acid have been evaluated [63]. Hence, support of research projects resulting in the proposal of novel, effective methods for the eradication of bacteria indexed by EPPO on A1 and A2 lists of pests recommended for the regulation as quarantine pests is of substantial importance.

In this case, the synthesized FRU-AgNPs were established to efficiently inhibit the growth of plant pathogenic bacteria cultivated in liquid media (Table 2). Interestingly, highly virulent Eam, Cm, and Xcc were shown to be more susceptible (MICs of 1.64 mg L^−1^) to the fabricated Ag nanostructures than the other species tested. On the other hand, Dsol showed the highest resistance with a MIC of FRU-AgNPs being 13.1 mg L^−1^. It is worth to notice that significant differences in the susceptibility of microorganisms from various species to AgNPs have been reported before [25,32,35]. Higher concentrations of FRU-AgNPs were needed to kill bacterial cells in comparison to inhibition of their growth as might be expected, with a notable exception of Rsol for which both MIC and MBC values were just 6.58 mg L^−1^ of FRU-AgNPs.

In our previous study, antibacterial properties of AgNPs stabilized either by sodium dodecyl sulphate (SDS) or pectins (PEC) were examined against bacterial phytopathogens belonging to the genera *Dickeya* and *Pectobacterium* [18] including the herein investigated Dsol IFB0099 strain. In reference to this bacterium, PEC-AgNPs and SDS-AgNPs showed higher effectiveness than FRU-AgNPs. Nevertheless, in order to inhibit the growth of Eam, Cm, and Xcc, lower MICs of FRU-AgNPs were needed than in the case of either SDS-AgNPs or PEC-AgNPs towards *Dickeya* or *Pectobacterium* strains (except for *Pectobacterium atrosepticum* IFB5103). In comparison to much more complex DNA-directed AgNPs grown on graphene oxide [24], FRU-AgNPs showed better performance against Xcc than the latter nanomaterial towards a closely related species such as *Xanthomonas perforans* (MIC 1.64 versus 16.0 mg L^−1^). The reported FRU-AgNPs were also more efficient against Xcc than the silica-silver of nanometric size [25] towards *Xanthomonas campestris* pv. *vesicatoria*. Taking into consideration that the mechanism of antibacterial action of AgNPs is not fully revealed yet [24], additional studies aimed at explaining how the genetic background contributes to the observed variation in the susceptibility of bacteria from different species to AgNPs are necessary. The latter research might provide further details on the interactions on the molecular level between AgNPs and the intracellular bacterial components.

## 4. Conclusions

Efficient, rapid, eco-friendly, and cost-effective synthesis of spherical and uniform FRU-AgNPs was accomplished by the incorporation of dc-APGD generated between the surface of the FLA solution (containing Ag(I) ions and d-fructose) and the pin-type W cathode in the continuous-flow reaction-discharge system. TEM and DLS measurements provided evidence that the produced Ag nanostructures were of a relatively small size and a narrow size distribution, i.e., 14.9 ± 4.3 and 15.7 ± 2.0 nm, respectively. The formation of metallic Ag of nanometric size was confirmed by SAED and EDX while functionalization of its surface by FRU was demonstrated by ATR FT-IR. Lastly, it was ascertained that FRU-AgNPs exhibit high antimicrobial activities against plant pathogenic bacteria subjected to expensive compulsory control measures under international legislation.

## 5. Patents

The method for the synthesis of metallic nanostructures is protected by Polish patent application No. P.417933.

## Figures and Tables

**Table 1:** Studied strains of bacterial phytopathogens and the utilized growth media.

Species, Abbreviation	Strain Nos ^a^	Disease Caused	Country, Year of Isolation	Host Plant	Growth Medium ^b^	Reference
*Erwinia amylovora*, Eam	IFB9037, CL0640	Fireblight	Poland, 2011	*Pyrus* spp.	Levan [48]	CL collection
*Clavibacter michiganensis*, Cm	IFB9038, CL0335	Bacterial canker	Poland, 2005	*Lycopersicon esculentum*	NCP-88 [49]	CL collection
*Dickeya solani*, Dsol	IFB0099, LMG28824	Blackleg, Soft rot	Poland, 2005	*Solanum tuberosum*	TSA (BTL, Poland)	[50,51]
*Ralstonia solanacearum*, Rsol	IFB8019, NCPPB4156	Brown rot	The Netherlands, 2001	*Solanum tuberosum*	TZC [52]	NCPPB collection
*Xanthomonas campestris* pv. *campestris*, Xcc	IFB9022, LMG582	Black rot	Belgium, 1980	*Brassica* spp.	GF [53]	[51,54,55]

^a^ IFB—Intercollegiate Faculty of Biotechnology University of Gdansk and Medical University of Gdansk (Gdansk, Poland), CL—Central Laboratory of Main Inspectorate of Plant Health and Seed Inspection (Torun, Poland), LMG—Belgian Coordinated Collections of Microorganisms (Gent, Belgium), NCPPB—National Collection of Plant Pathogenic Bacteria (London, UK). ^b^ To obtain the corresponding solid media, 15 g L^−1^ of agar was added.

**Table 2:** Minimal inhibitory and bactericidal concentrations of FRU-AgNPs against bacterial phytopathogens.

Bacterial Strain	MIC (mg L^−1^)	MBC (mg L^−1^)
Eam IFB9037	1.64 ± 0.05	3.29 ± 0.09
Cm IFB9038	1.64 ± 0.05	3.29 ± 0.09
Dsol IFB0099	13.1 ± 0.38	26.3 ± 0.75
Rsol IFB8019	6.58 ± 0.19	6.58 ± 0.19
Xcc IFB9022	1.64 ± 0.05	3.29 ± 0.09

Mean values ± standard deviations for three repetitions of the experiment are depicted.

## Abbreviations

AgNPs	silver nanoparticles
APM	atmospheric-pressure micro-plasma
APP	atmospheric pressure plasmas
ATR FT-IR	attenuated total reflection-Fourier transformation infrared spectroscopy
CL	Central Laboratory of Main Inspectorate of Plant Health and Seed Inspection
Cm	*Clavibacter michiganensis*
dc-APGD	direct current atmospheric pressure glow discharge
Dsol	*Dickeya solani*
DLS	dynamic light scattering
Eam	*Erwinia amylovora*
EDX	energy dispersive X-ray scattering
FAAS	flame atomic absorption spectrometry
FLA	flowing liquid anode
EPPO	European and Mediterranean Plant Protection Organization
FRU	fructose
FWHM	full width at half maximum
HV	high voltage
IFB	Intercollegiate Faculty of Biotechnology University of Gdansk and Medical University of Gdansk
LSPR	localized surface plasmon resonance
LMG	Belgian coordinated collections of microorganisms
MBC	minimal bactericidal concentration
McF	McFarland scale
MIC	minimal inhibitory concentration
NCPPB	National collection of plant pathogenic bacteria
NPs	nanoparticles
OD_600_	optical density at 600 nm
PEC	pectins
PLIs	plasma-liquid interactions
Rsol	*Ralstonia solanacearum*
RONS	reactive oxygen and nitrogen species
SAED	selected area electron diffraction
SDS	sodium dodecyl sulphate
TEM	transmission electron microscopy
Xcc	*Xanthomonas campestris* pv. *campestris*
